# Predictive research methods of enamel and dentine for initial caries detection

**DOI:** 10.1186/1878-5085-4-19

**Published:** 2013-06-26

**Authors:** Anatoly A Kunin, Irina A Belenova, Yury A Ippolitov, Natalia S Moiseeva, Dmitry A Kunin

**Affiliations:** 1 Therapeutic Dentistry Department, Faculty of Dentistry, Voronezh N.N. Burdenko State Medical Academy, Avenue of Revolution Str. 14, Voronezh, Russia

**Keywords:** Predictive Diagnosis, Initial Caries, Individual Prevention, Light-Induced Fluorescence, Electrometry

## Abstract

Currently, various research methods of enamel and dentine for precautionary diagnostics of initial caries forms are developed; however, the vast majority of these do not provide objective criteria of caries diagnostics or are very difficult to perform. Therefore, the search of diagnostics and enamel research methods, which will allow predicting caries emergence and to carry out personalised prevention of this pathology, is necessary. In this review, modern diagnostic methods that allow understanding the main aspects of caries process, assess the risk of its development, and also suggest the possibility of emergency prevention of caries progression in the nearest future are presented.

## Review

### Introduction

Recently, after the foundation of European Association for Predictive, Preventive and Personalised Medicine (EPMA), which includes 44 countries of Europe, Asia and other parts of the world, a definite tendency has been formed in ‘diagnostics’, defining the significant importance of preventive diagnostic methods and personalised prevention [1]. Similarly, methods to estimate the initial stages and forms of pathological processes of the teeth, gingival tissues, oral mucosa, joints and bones of the maxillofacial region have gained importance. Among all tissue types of both maxillofacial region and other body tissues, tooth caries has been shown to be the most frequent disease. Therefore, the registration of teeth lesions especially at the young ages will prevent the failure of the protective properties of the body and consequently will show the necessity of modifying socio-economical risk factors such as oral hygiene procedures, age-related factors, residence and dwelling place and social strata. In this review, the main preventing issues of tooth caries by detecting its early signs are presented which will help both dental professionals and experts on diabetes, oncology, haematology and endocrinology to potentiate the researches of hard dental tissues at the organ pathology level [2].

Comprehensive patient examination using the whole range of diagnostic methods is a crucial link for effective individual prevention of dental caries [3,4]. Diagnosis is defined as the section of medical science that outlines detection methods of different diseases and the patient's status in order to prescript the essential treatment and preventive measures [5]. Thus, the diagnosis in the narrow sense is patient examination for establishing of the disease diagnosis after the appearance of the initial symptoms. In contrast, individual caries prevention programme should be detached from the whole variety of methods since it should allow not only identifying available disease but also predict the pathology emergence or diagnose the beginning of illness at early preclinical stage, even when the pathology consecution have no clinical manifestations [6]. Accordingly, the programme of individual caries prevention is based primarily on data obtained during the diagnostic manoeuvres realisation, which allows predicting of caries. The determination of precarious status can be established by determining caries development signs and risk factors. Among the well-known and widely used methods in dentistry, it is necessary to allocate those that are capable to characterise the systematic relationship of ‘saliva-plaque-enamel’ as an important link in the dental caries pathogenesis [7,8].

Predictive diagnosis is a dynamic, multi-stage and multi-component process based on research results, and individual prevention is highly effective if it has etiopathogenetic character concerning each patient taken separately [9]. In our opinion, improvement of the practicability of methodological aspects of clinical diagnostics at individual caries prevention implementation is extremely important on the following positions:

1. Implementation stages of medical-diagnostic and preventive measures

2. The possibility of existence and development of dentition diseases requiring high-priority intervention and other specialist's consultation

3. Formation of a prognostic diagnosis (diagnoses) on the basis of experts in other sections of dentistry

4. Competent creation of health care activities

5. The necessity of the use of a significant amount of diagnostics methods for diagnostic inference verification

6. The choice expediency of rational prevention and medical-diagnostic ‘route’ and profiling of patients

7. Optimum sequence definition on the importance of performance degree of diagnostic and treatment measures

All currently known research methods and indexes, including hygienic, gingival, and caries prediction, have long been tested in practice, are informative in such area, and add up to a full-fledged clinical picture of the patient's dental status [10]. Unfortunately, performing the full range of proposed diagnostic techniques in practice is not possible due to the high cost and spending a considerable time interval as a whole. Dentists are faced in a situation of insoluble problem, where the easiest way is to cure a cavity, instead of looking for its causes and ways of risk factors elimination [11,12].

### Early caries forms detection methods and findings

The basis of tooth diseases diagnosing is the results summation of all its tissues studies, as well as other elements of dental-maxillofacial region, occlusion and articulation [13,14]. In other words, the diagnosis of ‘caries’ does not exclude the possibility of other maxillofacial diseases in patient and, therefore, does not relieve other physicians, which may seem unrelated at first glance, from the diagnosis and prevention of tooth caries, and once again emphasises the necessity of the individualisation of all measures held by the dentist [15].

Several studies [16-18] suggest that the initial demineralisation process of enamel occurring in the subsurface has no clinical manifestations which can be seen with the eyes and turns up as a preclinical stage of caries process development [19]. Thus, in our view, a special set of methods is necessary, including a variety of techniques, which characterises the hard dental tissues status at different precarious and caries stages [20,21]. For the purpose of timely diagnosis, prevention and personalised approach to the treatment of primary caries, a detailed study of the biochemical processes taking place in the structure of the tooth enamel and dentine in health and pathology is necessary [22,23].

#### Enamel apertures, tunnels, and bridges

**Figure 1:**
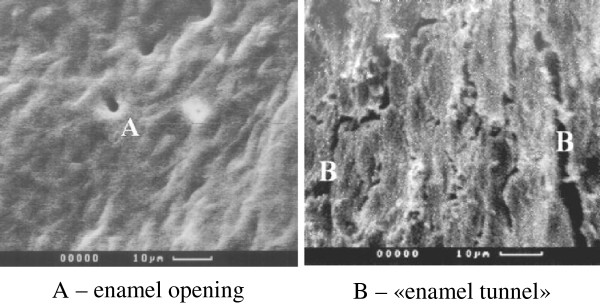
**Permanent tooth enamel.** (**A**) Enamel opening. (**B**) Enamel tunnel.

**Figure 2:**
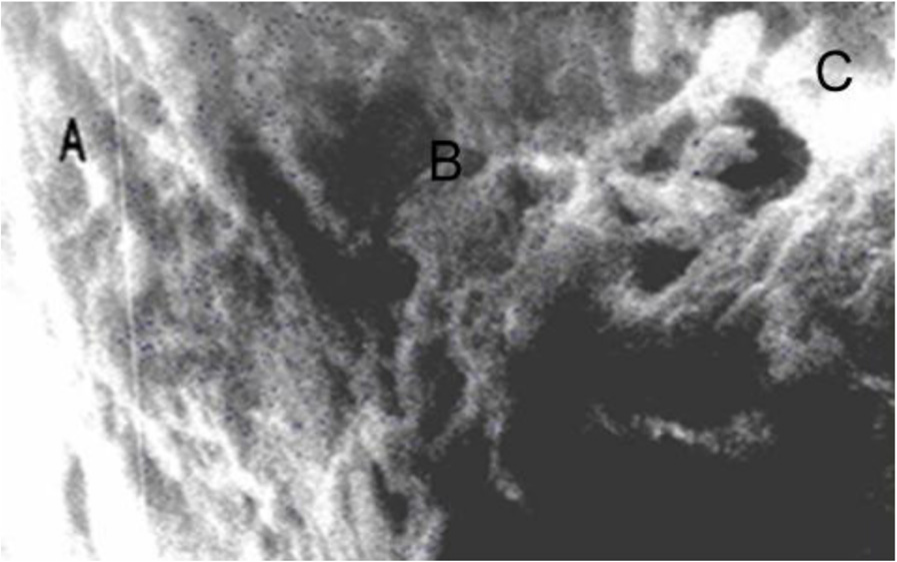
**Tooth slice, cementoenamel line (×5,000).** (**A**) Enamel, (**B**) enamel bridge and (**C**) dentin.

**Figure 3:**
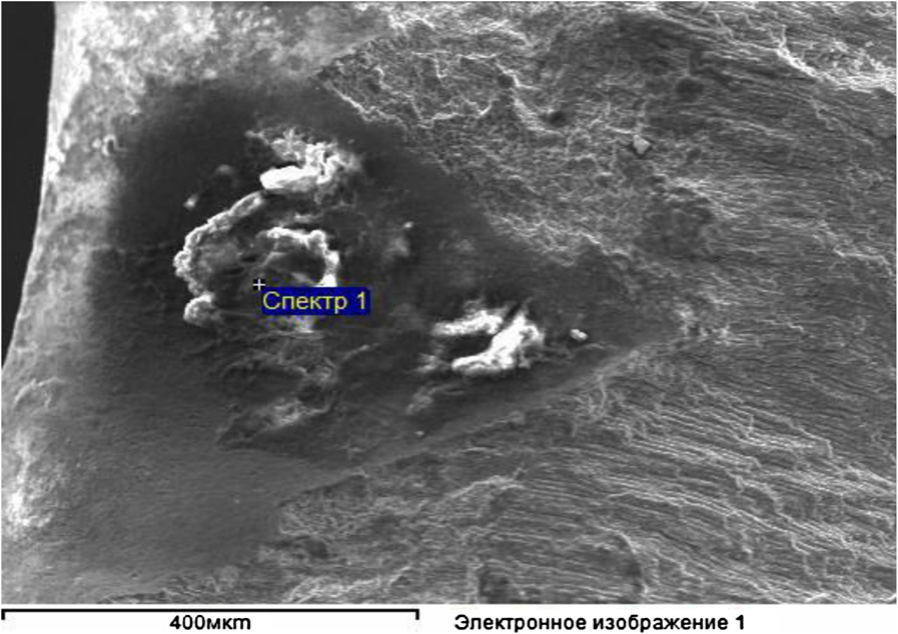
Enamel chip at subsurface demineralisation area.

In 1995 at the dentistry Department of Voronezh N.N. Burdenko State Medical Academy (VSMA), Professor A.A. Kunin et al. revealed new structural formations of enamel with the help of actual high-technology research methods. This previously unknown structure was the presence of apertures on the enamel surface, leaving inside the tooth in parallel of the enamel prism bunches [24]. Using scanning electron microscopy, the researchers established that these apertures penetrated through the dentine in some areas of enamel-dentine border [25]. The amount of such apertures in enamel surface at young age is high, but their quantity is reduced with the increasing age as a result of various damaging factors. Normally, these apertures have a diameter equal to 1 to 1.5 μm, increasing with age up to 3 μm (Figures 1, 2 and 3), with the reduction of their quantity [26]. Especially interesting is the identification of so-called ‘enamel bridges’ which are the penetrations of enamel ‘appendage’ into the dentine with preservation of ‘tunnel’ structure. In the presence of carie0073, these grooves or ‘bridges’ into the dentine can be broken; therefore, receiving mineral and organic compounds becomes complicated. There is only one access of necessary components for the preservation of tooth activity through a root canal [27].

There are numerous researches suggesting extensive information on the different changes in the tooth enamel. This information includes various data such as the initial changes existent in the surface structure of enamel and the parameters of the unit cell of enamel crystals [28,29]. However, none of these available data provide strong evidence on the importance of structures through the enamel.

#### Cariogenic plaque

The mechanism of the tooth metabolic processes in the demineralisation and remineralisation of enamel, which is the participation of enamel structures such as tunnels and bridges, is strongly affected by the activity of cariogenic plaque. Normally, the demineralisation and remineralisation processes are in a condition of dynamic balance in oral cavity, but in the presence of cariogenic factors, this balance shifts towards demineralisation [30,31]. The caries resistance of enamel depends on internal and external factors, but one of the main factors is the microbial dental plaque.

Today, the increased prevalence of caries and the high levels of cariogenic tooth plaque require individual approaches to the diagnosis and treatment of dental disease [32,33]. The high degree of cariogenic plaque subsequently leads to the formation of high-risk groups for caries development among the population of our country. Depending on the level of cariogenic plaque, the intensity of tooth plaque colouring by methylene red solution is determined according to a typographic scale as follows: 0 to 20, easy; 21 to 40, medium; and 41 to 60, heavy degree of cariogenicity. Thereby, the individual prevention directed on elimination of a cariogenic situation in an oral cavity is necessary [34-36].

#### Short-chain fatty acids

The pathogenic significance and physiological role of dental plaque should be considered to determine the correct strategy for its removal. Despite indisputable proofs of the infectious carious nature, infection realisation in carious process is not always observed. Therefore, the oral microflora can be divided into two categories: cariogenic and non-cariogenic. According to some scientists, ‘aggression’ of cariogenic bacteria is defined by a developing ecological situation in a dental plaque. The way of identification of a cariogenic and non-cariogenic tooth plaque in clinical practice is necessary [37,38]. The colonisation resistance of the oral mucous membranes is provided by the microflora metabolites, which include short-chain fatty acids (SCFA) such as acetic, propionic, isobutyric, isovaleric, valeric, izocapronic and capronic acid. SCFA, in parallel with the local immune system, play an important role in the stability resistance of the oropharynx epithelium, where the microflora of dental plaque has a specific contribution to the integral factors of bacterial metabolites in the oral fluid. The differentiation of the SCFA maintenance content in a tooth plaque is necessary for definition of enamel caries resistance. For this purpose, smears of tooth plaque are prepared on glass slide plates prepared by a drop of ferric chloride. After drying the glass over a torch flame (for the hydrolysis reaction of ferric acetate to acetic and propionic acids), the smear is evaluated by its colour, from brown (high content of SCFA) to light brown (low content of SCFA). For objective assessment of this method, dub preparation was subjected to microscopy investigation, digitalising the image using a video camera Sony MTV-62W1P (Minato, Tokyo, Japan) [39]. The optical density calculation of the dab preparation sites in the image analysis was performed using an image tool for Windows 3.0 with the Lambert-Bier formula. Previous researches stated that the reduction of the optical density of smears can signal the presence of caries. Depending on the optical density of smears obtained from the patient's plaque substrate, it is necessary to review the individual approach to oral hygiene [40].

Undoubtedly, the selection of individual hygiene means, taking into account particular properties of cariogenic situation in the mouth, will be a significant addition in the dental caries prevention. However, it is obvious that the problem of caries cannot be solved only with hygienic means [41]. The unity of enamel demineralisation and remineralisation mechanisms is that they are in dynamic balance, constantly occurring in-between oral liquid and enamel. The shift towards demineralisation means the beginning of the pathological structural and dynamic changes that occur in the enamel [42,43].

#### Organic and inorganic components of tooth

Some researchers [44-46] reasonably indicate an exclusive importance of the calcium-phosphorus correlation in the tooth enamel as well as the fact that the higher the value is, the longer it keeps steady crystal structure and resists influencing of acids; i.e., the Ca/P index is the criteria of tooth enamel stability, but there is no statement to what borderline value of the increase in Ca/P index promotes the gain of enamel resistance [47]. Currently, there are new data concerning the participation of enamel and dentine organic structure in the enamel metabolism, which finally lead to the emergence of clinically defined carious affection, depending on the formation rate and development intensity on specific features of an organism.

Due to the emergence of data on an organic component of tooth structure [48,49], the information of compound components and communication between the organic matrix and inorganic substances plays an important role in understanding of the processes occurring in hard dental tissues both in norm and at pathology [50]. Thus, carbohydrate and proteinaceous biopolymers and substance of protein nature objectively fill the interprismatic spaces and periodonto-blastic volume of dentinal tubules which are located in the tooth cement structures and also its soft fabrics, forming a physiological barrier to microorganisms and their metabolic products [51].

### Diagnostic methods

The abundance of methods of diagnostics and research to identify enamel caries has not yet yielded optimum results, which depends on individual approaches, and also on the importance of these methods in this particular case. Therefore, up to date, various techniques for the diagnosis of primary caries are suggested [52,53]; however, the vast majority of them do not provide objective criteria of firm fabrics defeat at caries or are very difficult to perform. We would like to allocate some of them in the first place.

#### Conductivity determination

The method of conductivity determination of enamel is intended to improve the reliability and objectivity of the initial caries recognition and also subsequent stages of the caries process [54]. Numerous clinical researches [55] have shown that the measurement of the electrical conductivity of hard dental tissues allows more accurate differentiation of healthy regions from those being struck with caries, excluding thereby, hyper diagnostics, preventing from excessive harmful intervention.

In 1990, Ivanova proposed a method for determining the electrical conductivity of hard dental tissues based on the measurement of size of the microcurrent passing through firm tooth fabrics on certain surfaces (the enamel splint, hillocks, fissures, borders of an abutment filling, dental plaque in cervical areas, vestibular surface and pulpless teeth), which allows to detect latent fissure, recurrent caries, and tooth caries at the ‘tooth-bracket’ border and filling attachment [56]. Electrical conductivity of hard dental tissues was determined by electro-diagnostic device (DENTEST, GeosoftDent, Moscow, Russia). Measurements were carried out at a constant voltage of 4.26 V, and the results of measurements were recorded in microamperes.

The method consists in careful drying of investigated teeth surface by air for 30 s. Passive electrode (dental surgery mirror) is placed in the oral cavity, providing thus good contact of it with soft fabrics of oral tissues. In microsyringe the active electrode gains an electrolyte solution (10% solution of calcium chloride) with glycerin so that at the end of the needle, the meniscus of electrolyte is formed. The active electrode establishes on carefully dried and examined tooth site and writes down instrument readings. This method allows for determining the density of the crystal lattice of tooth enamel, which increases at enamel demineralisation with resistance loss. In its basis lie the inverse relations of tooth conductivity from mineralisation level of its fabrics. This method allows simple and accurate diagnosis of the initial latent caries, which confirms the clinical relevance of its usage in dentistry.

#### Light-induced fluorescence

The search for diagnostics and enamel research methods which can further estimate the data obtained at an assessment of conductivity is necessary, which include light-induced fluorescence. The basis of this caries diagnosis method is the phenomenon of porphyrin fluorescence - waste products of cariogenic microflora - under the influence of a laser or visible light of a certain wavelength [57-59].

In the method of laser fluorescence, a laser diode is used as a power source with a wavelength of 655 nm and light capacity equal or less than 1 MW. DIAGNOdent (KaVO Dental, Charlotte, NC, USA) is one of these devices with the same parameters. This device is used for the diagnosis of initial caries of fissures and vestibular surfaces of the tooth; however, the high price of the device and the complexity of the procedure (tooth preparing-cleaning before the diagnostic procedure) limit its routine use [60,61].

For an assessment of light-induced fluorescence of hard dental tissues and primary dental caries diagnosis, we propose to use the domestic light-emitting-diode activator ‘LED active’, (MEDTORG+, Kirov, Russia), with a wavelength of 530 nm and illumination of 10,000 metre-candle, and also wavelength of 625 nm at the 140 MW/cm^2^ radiation power density. The activator's principle action is based on the application of light powerful light-emitting diodes with the big intensity of a luminescence of monochrome colour without a thermal component [62,63].

At an inspection of smooth surfaces of enamel or visible cement of a root by means of radiation of green colour, the centre’s initial demineralisation, in the form of change of fluorescence in the defeat centre, is most effectively diagnosed [64]. Using light radiation at inspection in determining fluorescence parameters, occlusal surfaces change to red colour long before demineralisation occurs and products of a metabolism of microorganisms are produced. The above metabolism of microorganisms in the centre of demineralisation differs in luminescence from the fluorescence of healthy fabrics [65]. Clinically, light-induced fluorescence can be used for a choice of preventive tactics concerning tooth, as it allows distinguishing between healthy teeth and teeth with low and high risk of caries [66].

Further researches of hard dental tissues structural change in preclinical and early caries stages using novel methods, based on new physical approaches namely, the light-induced fluorescence and electrometry [67-69], are necessary in order to carry out large-scale researches on the prevention, early diagnosis and personalised treatment of caries and identification of the pathogenesis of its development and progression. In our opinion, the use of these techniques is essential for early diagnosis and prevention of tooth caries, which will significantly improve the accuracy and timeliness of diagnosis statement, and also the quality of treatment and preventive process. Comparison of the characteristics of clinical and laboratory parameters of intact enamel and at initial caries stage by current diagnostic methods is important, which will allow to predict emergence of caries and to carry out purposeful prevention of this pathology [70-72].

#### X-rays

If to mean preclinical diagnostics of the initial caries stage, there are methods capable ‘to catch’ demineralisation signs. These methods have to state remineralisation effect and confirm a complete enamel recovery, thereby optimising the development of remineralising therapy methods [73-75]. The above methods estimate them, but the most important thing is the determination of how the remineralisation occurs.

The most practiced and most reliable method is X-ray in identifying latent cavities by passing rays [76]. The identification of preclinical symptoms of caries is problematic today. Doctors now in most cases do not estimate the initial extent of demineralisation on the X-ray image though, if increased, is probably possible [77]. As a result of complex application of intraoral radiography, caries stages can be evaluated by five points with light-induced fluorescence and measurements of electric resistance of hard dental tissues [57].

#### IKLORZ index

Assessment of the clinical and laboratory evaluation of resistance of hard dental tissues (IKLORZ) [78] is defined as the relation of the sum of the caries points to the total number of examined teeth, and this ratio is calculated using the formula

$$I_{\mathrm{IKLORS}}=\frac{\sum R1+\sum R2+\sum R3+\sum R4}{32},$$

where *I*_IKLORS_ is the index of clinical assessment of hard dental tissues; *R*0 is the caries at white spot stage; *R*1 is the superficial caries 1; *R*2 is the superficial caries 2; *R*3 is the average caries; *R*4 is the deep caries.

So for example, in an examination of a patient with 32 teeth, if there are fissure caries within enamel of two molar teeth and initial caries on three teeth in the form of a white spot, the calculation of a clinical assessment IKLORS index of firm fabrics status in this patient shows

$$I_{\mathrm{IKLORS}}=\frac{2+2+1+1+1}{32}=0.22.$$

Thus, the index of clinical assessment of hard dental tissues of the patient reaches the value of 0.22.

The given researches convincingly show that increase of an index value through a certain period refers to an unfavourable carious gain in oral cavity and index reduction is related to adequate treatment and preventive measures [30].

### Properties of an ideal diagnostic caries technique

The search of new highly effective diagnostic and preventive techniques at initial caries of teeth is, today, a priority problem of precautionary and preventive dentistry [79-81]. The important point is the selection the most informative and important method among the common list of diagnostic tests. By exploring all possible ways of improving the caries diagnosis, we came to the conclusion that in this case, the most urgent is the development of an automated procedure based on mathematical methods for solving problems. This procedure must include the following:

1. The totality formation of clinical caries signs towards the selection of most informative indicators with the highest diagnostic value and determination of their sequence on practical reception.

2. The allocation of the main clinical and anamnesis indicators which promote caries disease in order to form groups of patients [82-84].

To develop the most complete list of indicators that predicts the occurrence of caries process, we used the data of scientific, methodological literature and collaboration of the Therapeutic Dentistry Department staff. Thus, the list of symptoms of potentially possible caries was finally created [85]. We have referred the following diagnostic receptions to methods of caries forecasting (Table 1).

**Table 1:** Forecasting methods of caries and identifying factors inspiring its emergence

**Number**	**Method**	**Structure/component**	**Factor that potentiate caries development**
1	Polling	-	-
2	Inspection, probe	-	-
3	Definition of the caries intensity	Hard dental tissues	Caries susceptibility of enamel and dentine
4	Definition of the plaque quantity	Teeth enamel	Local enamel demineralisation, retrogression of oral fluid remineralising ability
5	Definition of plaque formation rapidity	Teeth enamel	Local enamel demineralisation, retrogression of oral fluid remineralising ability
6	Definition of tooth plaque cariogenicity	Teeth enamel	Local enamel demineralisation, retrogression of oral fluid remineralising ability
7	Definition of enamel functional status (ТER test)	Teeth enamel	Level of enamel caries’ susceptibility
8	Clinical evaluation of remineralisation speed in enamel (CDERS test)	Teeth enamel	Level of enamel caries’ susceptibility
9	Acidic enamel biopsy	Teeth enamel	Level of enamel caries’ susceptibility
10	X-ray spectrum microanalysis	Teeth enamel and dentine	Low level of macroelements and/or microelements, influence on enamel caries’ resistance
11	Electric pulp test	Teeth pulp	Rise or fall of pulp affectability and its influence on enamel caries’ resistance
12	Electrometric definition of hard dental tissues	Teeth enamel and dentine	Reduction of hard dental tissues microhardness and its influence on enamel caries’ resistance
13	Revelation of enamel demineralisation centres on the border with filling material	Teeth enamel	Revelation of caries liability at the enamel region
14	Definition of unsatisfactory filling	Teeth enamel	Revelation of caries liability at the enamel region
15	Definition in periodontal treatment requirement	Periodontal tissues	Local variation of рН caused by gingival fluid, bacterial component in oral cavity, and exponentiation of caries development
16	Definition of gingiva inflammation level	Periodontal tissues	Local variation of рН caused by gingival fluid, bacterial component in oral cavity, and exponentiation of caries development
17	Definition of gingiva angiostaxis degree	Periodontal tissues	Local variation of рН caused by gingival fluid, bacterial component in oral cavity, and exponentiation of caries development
18	Definition of combined saliva viscosity	Oral fluid	Reduction of enamel caries resistance, remineralising ability of oral fluid
19	Definition of combined saliva acidity	Oral fluid	Reduction of enamel caries resistance, remineralising ability of oral fluid
20	Definition of salivation rapidity	Oral fluid	Reduction of enamel caries resistance, remineralising ability of oral fluid
21	Definition of status of buffered saliva characteristic	Oral fluid	Reduction of enamel caries resistance, remineralising ability of oral fluid
22	Definition of oral fluid microcrystallisation exponent	Oral fluid	Reduction of enamel caries resistant, remineralising ability of oral fluid
23	Bacterioscopy	Microflora of oral cavity	Violation of bacteritic balance in oral cavity, activation of tentatively pathogenic and pathogenic microorganisms, reduction of enamel caries resistance
24	Cytology	Microflora of oral cavity	Violation of bacteritic balance in oral cavity, activation of tentatively pathogenic and pathogenic microorganisms, reduction of enamel caries resistance

Submitted list of diagnostic methods allows understanding the main aspects of caries process, assesses the risk of its development, and also suggests the possibility of emergency prevention and/or caries progression in the nearest future [86-88].

However, the usage by dentist of such a large range of 24 forecasting methods, primarily qualitative type, is not conducive to effective diagnostic and therapeutic processes in caries prevention because of the following: firstly, the usage of a large number of indicators complicates work at the practical appointment in the diagnosis statement; secondly, in our view, it is a kind of diagnostic liability and not only raises up the reliability of diagnosis, but apparently in some cases even reduces it; thirdly, some data duplicate each other, describing the same sign of tooth caries.

In this paper, to assess the 24 signs of predicting caries and to highlight the most significant of these, we used the method of a prior decent ranking, which allows to objectively assess the subjective opinions of specialists (experts) [89-91]. Leading specialists from the Therapeutic Dentistry Department of VSMA in N.N. Burdenko, who have already had a doctorate degree or PhD in dealing with theoretical and practical problems of caries pathology and with work experience from 15 to 38 years, have been recruited to the expert committee. Fifteen experts were asked to complete a questionnaire, ranking the techniques where the caries susceptibility rates were assessed by the level of their influence, decrease on the emergence and development of the pathological process, and also to place these techniques in sequential order while examining the patient. Dichotomy (sequential decomposition of disease signs hyperspace in two areas by cutting planes) was applied for improving the efficiency of ranking method. Thus, the whole summation of predictive caries signs was divided by each of the experts into two groups, one of which, according to experts, would consist of parameters, which in a greater degree of influence on the caries process occurrence, and the second, from less affected. Then, each group of indicators again was divided into two groups, etc. - as long as the number of features was not more than five. In this case, ranking the options in each group and overall was more genuine. In accordance with the parameter importance, its place in the sequence of diagnostic procedures was determined [92,93].

**Figure 4:**
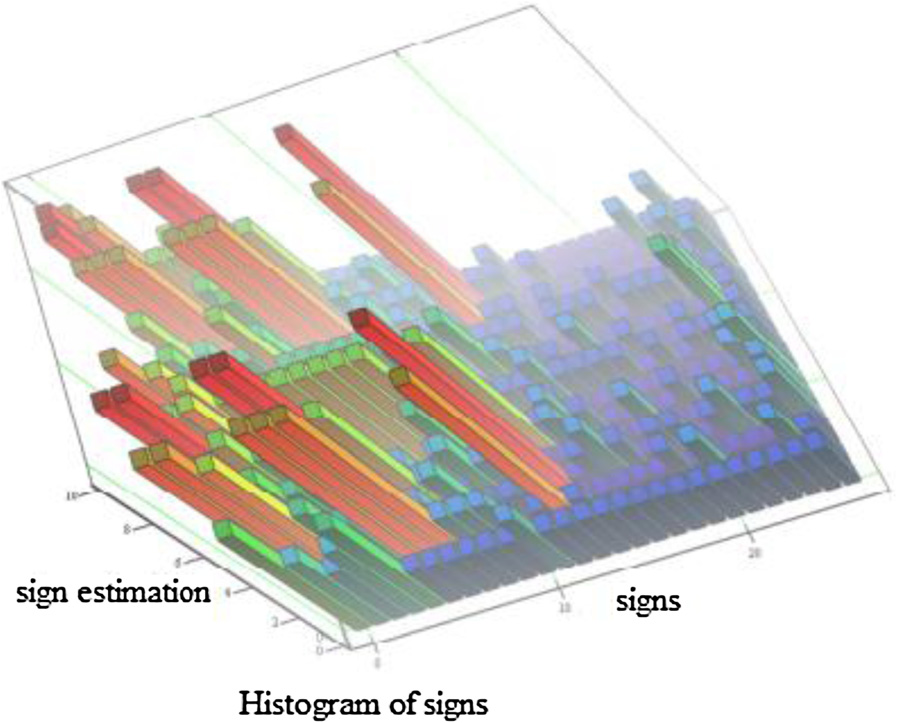
Ranking histogram.

By the combination of the experts’ opinion, a matrix of rank was formed, containing the matching rank. According to research results, a ranking histogram was built (Figure 4), where the horizontal axis plotted the corresponding numbers of diagnostic methods of caries prediction in descending order according to the degree of its self-descriptiveness and the ordinate for each indicator delayed generalised rank-sum value, which essentially characterises the measurement consistency of expert physicians in assigning to this parameter-given rank.

The histogram shows that there is an uneven distribution of signs and decrease in the factors influence which is not homogeneous and therefore, the most essential factor may be separately analysed. Thus, according to the experts, from the 24 indicators, 14 that are more informative are shown in Table 2 in order which, in the expert committee opinion, is the most preferred for dental examination.

**Table 2:** List of the main caries prediction methods and sequence of conducting dental examination

**Number**	**Method**	**Structure**
1	Polling	-
2	Inspection, probe	Oral cavity
3	Definition of the caries intensity	Dentition
4	Definition of hydrogen ion exponent in oral fluids	Oral fluid
5	Definition of hygienic status of the oral cavity	Teeth enamel
6	Definition of the tooth plaque cariogenicity	Teeth enamel
7	Acidic enamel biopsy	Teeth enamel
8	Clinical evaluation of remineralisation speed in enamel (CDERS test)	Teeth enamel
9	Definition of enamel functional status (ТER test)	Periodontal tissues
10	Definition of gingivitis index	Teeth enamel
11	Bacterioscopy	Mucous membrane of oral cavity
12	Cytology	Mucous membrane of oral cavity
13	Revelation of enamel demineralisation centres on the border with filling material	Teeth enamel and dentine
14	Electrometric definition of hard dental tissues	Teeth enamel

## Conclusions

In conclusion, the list of the most informative prognostic caries criteria included those signs, which are often met and the identification of these do not pose a significant challenge for the dentist. It is possible to use the highlighted results of the most significant predicting caries factors for (a) targeting specification of patients' dental status and its distribution to the prevention groups; (b) planning, based on the indicators data, individual preventive measures, preventing caries process; and (c) assessing the effectiveness of prevention programmes and their timely correction [94-96].

## Competing interest

The authors declare that they have no competing interests.

## Authors’ contributions

AAK carried out the molecular genetic studies, participated in the sequence alignment, conceived the study, participated in its design and coordination, and drafted the manuscript, IAB participated in the sequence alignment and designing of the study, and performed the statistical analysis. YAI carried out the molecular genetic studies, participated in the sequence alignment, and drafted the manuscript. NSM also participated in the sequence alignment. DAK carried out the molecular genetic studies, participated in the sequence alignment, and drafted the manuscript. All authors read and approved the final manuscript.

## Author’s information

AAK is a coordinator of specialised section in dentistry (DPPPD) of EPMA.
